# Increased serum microRNAs are closely associated with the presence of microvascular complications in type 2 diabetes mellitus

**DOI:** 10.1038/srep20032

**Published:** 2016-02-01

**Authors:** Cheng Wang, Shujun Wan, Ting Yang, Dongmei Niu, Aisen Zhang, Cuihua Yang, Jialu Cai, Jia Wu, Jiaxi Song, Chen-Yu Zhang, Chunni Zhang, Junjun Wang

**Affiliations:** 1 Department of Clinical Laboratory, Jinling Hospital, Medical School of Nanjing University, Nanjing, 210002, China; 2 State Key Laboratory of Pharmaceutical Biotechnology, Nanjing Advanced Institute for Life Sciences, Nanjing University School of Life Sciences, Jiangsu Engineering Research Center for MicroRNA Biology and Biotechnology, Nanjing University, Nanjing, 210046, China; 3 Department of Geratology, the First Hospital Affiliated to Nanjing Medical University, Nanjing, 210029, China; 4 Department of Endocrinology, Jinling Hospital, Medical School of Nanjing University, Nanjing, 210002, China

## Abstract

Circulating microRNAs (miRNAs) are emerging biomarkers for type 2 diabetes mellitus (T2DM). However, a comprehensive characterization of the serum miRNA profile in patients with T2DM-associated microvascular disease (T2DMC) has rarely been reported. In this study, we obtained serum samples from 184 T2DM patients (92 with microvascular complications and 92 free of complications) and 92 age/gender-matched controls. The levels of 754 miRNAs were initially analyzed using a TaqMan Low Density Array (TLDA) in three pooled samples from 24 T2DM patients, 24 T2DMC patients and 24 controls. Markedly upregulated miRNAs in the patients’ groups were subsequently validated individually by quantitative reverse-transcription PCR (RT-qPCR) in the same samples used for TLDA and further confirmed in another larger cohort consisting of 68 patients with T2DM, 68 patients with T2DMC and 68 controls. Five miRNAs were significantly upregulated in T2DM patients (p < 0.05) including miR-661, miR-571, miR-770-5p, miR-892b and miR-1303. Moreover, the levels of the five miRNAs were higher in patients with complications than in those without complications. Regression analyses revealed the five miRNAs were significantly correlated with microvascular complications (p < 0.05). The five serum miRNAs identified in our study hold potential as auxiliary biomarkers and novel risk factors for T2DM-associated microvascular complications.

Type 2 diabetes mellitus (T2DM) is a chronic metabolic disease that is characterized by high blood glucose levels, relative insufficiency of insulin secretion from pancreatic beta cells and insulin resistance^1^. T2DM has become a major global health challenge. Recent epidemiological research has demonstrated that T2DM prevalence for all age groups has increased sharply in the last decade, rising from 171 million in 2000 to 336 million in 2011. Even worse, the number of T2DM patients is expected to reach 400 million worldwide in the next few decades^2^,^3^. In adults, T2DM patients with prolonged high blood glucose levels often demonstrate microvascular complications including neuropathy, nephropathy and retinopathy, which are the most common causes of blindness, nontraumatic amputations and renal insufficiency disease^4^,^5^. Consequently, it is important to recognize and treat this devastating disease early in its progression to postpone or even prevent the serious complications that are associated with it. To date, several risk factors have been identified that implicate microvascular complications, such as advanced glycation end products, inflammatory cytokines and increased levels of oxidant stress^6^,^7^. Nevertheless, the major mechanisms underlying the T2DM related microvascular complications remain elusive. Moreover, reliable biomarkers for long-term microvascular complications in T2DM are also lacking. Hence, there remains a critical need to better understand the underlying disease mechanisms that are responsible for diabetic complications to develop new and improved therapeutic strategies for these chronic conditions.

Recent studies have uncovered a novel group of universally present small non-coding RNAs known as microRNAs (miRNAs), which are emerging as pivotal regulators for various biological processes and disease progression^8^. Furthermore, miRNAs also play critical roles in glucose (GLU) homeostasis and T2DM pathogenesis. Currently, a vast number of miRNAs have been implicated in pancreatic development, insulin secretion, and beta cell dysfunction^9^. Beyond hyperglycemia, miRNAs also participate in the inflammatory response, as well as vascular endothelia damage and fibrosis processes, all of which are major and well-known hallmarks of diabetic complications^10^. It is therefore not surprising that miRNAs also contribute to the occurrence and development of common microvascular complications. Indeed, several existing lines of evidence have demonstrated that T2DM patients with diabetic complications are often associated with certain dysregulated miRNAs in various target tissues, especially the brain, eyes, nerves and kidneys. Studies from our group and others have recently highlighted that miRNAs are also stably detectable in the circulation and can be used as potential non-invasive biomarkers for various diseases, including T2DM^11^. However, most existing studies are based on a limited number of patients or only focus on a few specific miRNAs, which may partially explain some of the discrepancies observed^12^,^13^. Systematic analyses of serum miRNAs in T2DM and T2DM with microvascular complications have rarely been performed.

Therefore, in the present study, we aimed to systematically and comprehensively evaluate miRNA expression patterns in serum samples of T2DM patients and T2DMC patients as well as non-diabetic individuals. To relate these findings to the pathogenesis of T2DM and T2DMC, we performed a reverse transcription RT-PCR-based TaqMan Low Density Array (TLDA) combined with confirmation by quantitative reverse-transcription PCR (RT-qPCR). Our results successfully identified five serum miRNAs, including miR-571, miR-661, miR-770-5p, miR-892b and miR-1303 that were significantly increased in T2DM patients; furthermore, the levels of these five miRNAs were markedly higher in patients with microvascular complications than in those without complications.

## Results

### TLDA analysis of serum miRNAs in T2DM with and without microvascular complications

**Figure 1:**
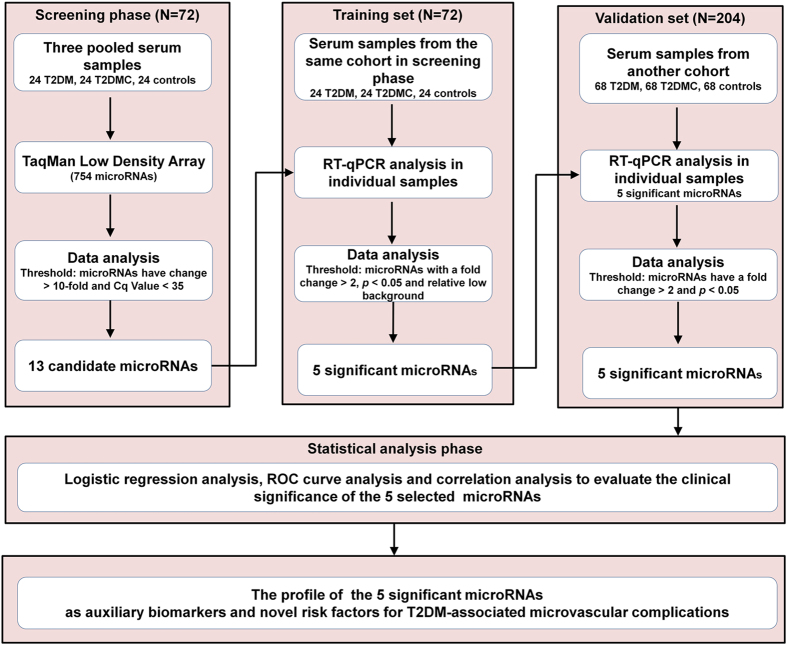
Study design. T2DM, type 2 diabetes; T2DMC, type 2 diabetes with microvascular complications; Cq, quantification cycle; RT-qPCR, quantitative reverse transcription polymerase chain reaction; ROC, receiver operating characteristic.

To select miRNAs that are present at different levels in T2DM patients with and without microvascular complications and in non-diabetic control individuals, we initially employed the RT-qPCR based TLDA chip to analyze global miRNA profiles in three pooled serum samples from 24 patients with T2DM, 24 patients with T2DM microvascular complications and 24 healthy controls (Fig. 1). The results revealed that the serum miRNA expression profiles varied among the T2DM patients and the healthy controls. Pearson correlation and scatter plot analyses revealed that the correlation coefficients (R^2^) between the two T2DM patient groups and the controls are relatively low; the R^2^ value was 0.68 between the T2DM patients and the non-diabetic controls and 0.67 between the T2DMC patients and the non-diabetic controls (Supplementary Fig. S1A, B). Moreover, we also analyzed the association between the two patient groups, and the Pearson correlation R^2^ value for the two groups was 0.76, which suggested a common but different serum miRNA expression profile (Supplementary Fig. S1C). miRNAs were considered to be upregulated if their Cq values were <35 in the serum samples of both the two T2DM groups patients and downregulated if their Cq values were <35 in the control samples and if their serum production had at least a 2-fold alteration in both T2DM patient groups compared with the control samples. Of the 754 miRNAs assessed, 25 were upregulated and 118 were downregulated in the two T2DM patient groups compared with the non-diabetic controls. For the dysregulated miRNAs, 13 miRNAs that were markedly increased in the two T2DM patient groups with fold change >10 in the T2DMC group were selected for further RT-qPCR validation with individual serum samples and are listed in Table 1.

### Confirmation of increased serum miRNAs by individual RT-qPCR analysis

To verify the TLDA results, we next performed RT-qPCR analysis for the 13 markedly upregulated miRNAs in the same cohort of patients whose serum samples were pooled for TLDA. The clinical features of the participants of the two groups of patients and controls are listed in Table 2. As can be seen in Table 2, patients with T2DM and T2DM associated microvascular disease have a markedly higher body mass index (BMI), systolic blood pressure (SBP) and diastolic blood pressure (DBP) than the control subjects (p < 0.05). Furthermore, serum total cholesterol (TC), triglyceride (TG) and low-density lipoprotein-cholesterol (LDL-C) concentrations were also significantly elevated in both T2DM patient groups as compared with the controls, while serum high-density lipoprotein-cholesterol (HDL-C) levels were markedly decreased (p < 0.05).

**Figure 2:**
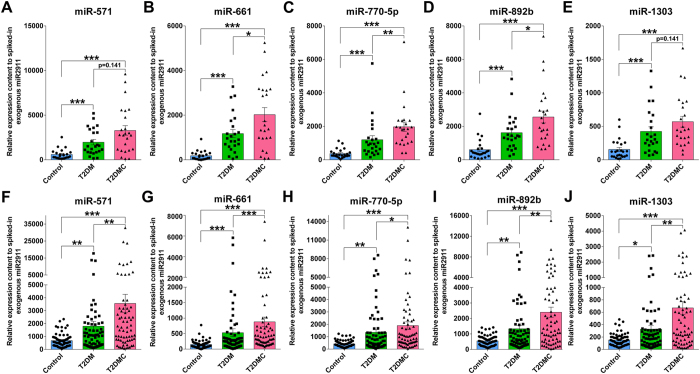
Relative levels of the five identified serum miRNAs in the T2DM, T2DMC and non-diabetic control groups of training set (A–E) and validation set (F–J). Cq values were converted to relative concentrations normalized to MIR2911 values and were calculated using the comparative Cq method (2^−ΔCq^). Each point represents the mean of triplicate samples. *p < 0.05; **p < 0.01; ***p < 0.001.

The inclusion criteria for significantly upregulated miRNAs that were chosen from the training set for further validation were a mean fold change >2 and a p-value < 0.05 between the two patient groups and control group, and the difference in the Cq value of the candidate serum miRNA between the control group and negative control (PCR with no template RNA, referred as “background noise”) was at least >3 cycles. In addition, miRNAs with a Cq value >35 and a detection rate <50% in the patient or control groups were also excluded from further analysis. Using these criteria, the levels of five miRNAs including miR-661, miR-571, miR-770-5p, miR-892b, and miR-1303 were significantly increased in both the T2DM patients with and without complications relative to healthy controls (Fig. 2 and Supplementary Table S1). Furthermore, we observed that the levels of the above five serum miRNAs were higher in T2DMC patients than in those who were free of complications (Fig. 2), whereas there was no marked difference in glucose levels and serum lipid parameters between the two subgroups (Table 2).

To further assess the alteration of these five serum miRNAs as a signature of T2DM and T2DM microvascular complications, we assessed the levels of the above-identified five miRNAs in another larger independent cohort that was referred to as a validation set and that included 68 T2DM patients, 68 T2DMC patients and 68 controls. As shown in Fig. 2, changes in levels of the five miRNAs in the two case groups were consistent with the results from the training set.

The relative levels of these five serum miRNAs in all the patients and the control individuals that enrolled in the training and validation sets were shown in Supplementary Fig. S2. Consistent with our expectations, the concentrations of all 5 miRNAs were significantly increased in both the two T2DM patients’ groups, as compared with control group. Notably, the difference in the levels of these miRNAs between the case groups was more marked (Supplementary Fig. S2). In addition, we also calculated the absolute concentrations of the five selected miRNAs by conducting calibration curves developed with corresponding synthetic mature miRNA oligonucleotides (Supplementary Fig. S3), since this approach has been proved to be greatly successful in identifying serum miRNA-based biomarkers in our previous studies^14^,^15^,^16^. As shown in the Supplementary Fig. S4, all the five miRNAs showing the same tendency as normalizing to spiking-in exogenous MIR2911, which were further confirmed our results.

### Validation of T2DM-associated miRNAs in serum samples

It is notably that there is no overlap between miRNA changes in T2DM and T2DMC observed by previous studies^12^,^13^ and changes in serum miRNAs found in the current study. In order to test the reliability and reproducibility of the patients as well as the methodology applied in this study, we then measured the serum levles of six miRNAs including miR-15a, miR-16, miR-125b, miR-126, miR-221 and miR-320a that were selected from literatures^12^,^13^ and related with T2DM and T2DM complications in the serum samples of validation set in our present study, including 31 normal controls, 31 T2DM patients and 31 T2DMC patients. As showed in the Supplementary Fig. S5, all the six miRNAs were highly expressed in T2DM and T2DMC patients as compared with controls, which were consistent with previous studies except for miR-126. Interestingly, of the six examined miRNAs, three (miR-125b, 126 and 221) were significantly elevated in T2DM patients, while four (miR-16, 125b, 126 and 221) were significantly elevated in T2DMC patients (p < 0.05). These reproductive results demonstrated that the patients and methods applied in this study is reliable and convincible.

### Characterization the relative concentrations of the selected serum miRNAs in T2DMC patients caused by different etiology

Because we enrolled a very mixed population represented by T2DMC patients with diabetic foot, retinopathy, neuropathy, nephropathy and a combination of the above, we then explore the difference in the levels of the five selected miRNA in the subtypes T2DMC patients caused by different pathogens. As shown in the Supplementary Fig. S6, when compared the serum levels of the five selected miRNAs in T2DMC patients represented with different conditions, we observed that there was no significant difference between each patients groups, while the concentrations of the five miRNAs in T2DMC patients with different subtypes showing significant higher levels than the healthy control subjects except for in the two patients with diabetic foot. These results may partially prove that the alterations of the five miRNAs in serum from T2DMC patients were not introduced by the different clinical conditions but naturally resulted from some common mechanisms.

### Univariate and multivariate logistic regression analysis

We next performed a forward stepwise binary logistic regression to weigh the usefulness of the selected miRNAs in T2DM subjects using the status of T2DM with and without microvascular complications as the dependent variable. Using the control group as the reference category, when the T2DM with or without microvascular complications disease group was treated as a dependent two-category variable, the selected five serum miRNAs were independently correlated with simple T2DM and T2DMC. The odds ratios (ORs) of these miRNAs for T2DM were as follows: miR-571 (OR = 7.6, 95% CI, 2.8–20.8, p < 0.001), miR-661 (OR = 11.2, 95% CI, 4.1–30.2, p < 0.001), miR-770-5p (OR = 9.7, 95% CI, 3.6–26.4, p < 0.001), miR-892b (OR = 8.0, 95% CI, 2.9–21.8, p < 0.001) and miR-1303 (OR = 5.5, 95% CI, 2.0–15.2, p < 0.001). For T2DMC group, the ORs were as follows: miR-571 (OR = 19.8, 95% CI, 7.4–53.4, p < 0.001), miR-661 (OR = 25.9, 95% CI, 9.6–69.8, p < 0.001), miR-770-5p (OR = 22.6, 95% CI, 8.4–60.9, p < 0.001), miR-892b (OR = 19.8, 95% CI, 7.4–53.4, p < 0.001) and miR-1303 (OR = 16.7, 95% CI, 6.2–44.8, p < 0.001) (Table 3). To further evaluate the associations of these serum miRNAs for categories of microvascular complications, we found all the five miRNAs were independently associated with microvascular complications when the T2DM group was treated as the reference category (Table 3). These results suggest that these five miRNAs are potent risk factors for the presence of T2DM associated microvascular complications.

Furthermore, after adjusting for age, gender, BMI, smoking status, alcohol consumption habits and blood pressure status, miR-661 and miR-892b still remained an independent association with T2DM, and miR-661 and miR-571 demonstrated an independent association with T2DMC by multivariate logistic regression analyses using a dependent two-category variable (Table 3).

To further evaluate the predictive usefulness of the combination of the five selected miRNAs, we conducted a risk score analysis to construct a signature using these five miRNAs. Each patient or control was assigned a risk score based on a linear combination of the levels of the five miRNAs weighted by their regression coefficients as previously described^14^,^15^,^16^. L ogistic regression demonstrated that the OR of the five-serum miRNA panel was 7.2 (95% CI = 3.5–14.8, p < 0.001) for T2DM, 16.3 (95% CI = 7.7–34.3, p < 0.001) for T2DMC, and 2.3 (95% CI = 1.2–4.2, p = 0.01) for distinguishing T2DMC from T2DM (Table 3). These results indicated that the five identified miRNAs and their panel enable the detection of T2DM and T2DM-associated microvascular complications and may be useful as novel risk factors for T2DMC, further serving as valuable new blood parameters that will help physicians refine early therapeutic interventions.

### ROC curve analysis

To evaluate the association of the five altered serum miRNAs with T2DM with and without microvascular disease, we performed receiver operating characteristic curve (ROC) analysis on the selected miRNAs in all of the patients and controls enrolled in this study and yielded area under ROC curve (AUCs) from 0.67 to 0.74 for the T2DM and control groups and from 0.79 to 0.87 for the T2DMC and control groups (Supplementary Table S2). Furthermore, ROC curves were also constructed to evaluate the discriminative ability of the five miRNA profiles for the T2DM and T2DMC group, and AUCs were from 0.62 to 0.65 (Supplementary Table S2). Moreover, based on their risk scores of patients and control samples, we further evaluate the diagnostic value of the five-miRNA profiling system and found the AUC for the combination of the five miRNAs was 0.71 (95% CI = 0.64–0.79, p < 0.001) and 0.83 (95% CI = 0.77–0.89, p < 0.001) for the T2DM and T2DMC patients, respectively. In addition, the AUC is 0.65 (95% CI = 0.57–0.73, p = 0.001) for discriminating T2DMC patients from T2DM patients (Supplemental Table S2). Taken together, these results indicated that the five miRNAs has a relative high diagnostic value for T2DMC.

### Association of serum miRNA levels with clinical parameters

We subsequently wondered whether serum miRNA expression levels were correlated with serological biochemical parameters. We evaluated the associations between the clinical features and miRNA abundance using Spearman rank correlation analysis in all of the studied individuals. As demonstrated in Supplementary Table S3, all five miRNAs had strong positive correlations with GLU and TG (p < 0.05) and significantly negative relationships with HDL-C (p < 0.05) (Supplementary Table S3). Together with the RT-qPCR results, these data suggest that the upregulation of these five miRNAs in T2DM patients may be involved in T2DM pathogenesis and diabetic microvascular complications.

## Discussion

To date, nearly 10 pioneering studies have characterized and defined specific signatures of serum, plasma or blood cell miRNA profiles in an attempt to develop new approaches to predict and monitor T2DM development and progression^17^. These studies have raised the intriguing possibility of and led to rapid progression in using circulating miRNAs as novel biomarkers for T2DM disease. However, systematic analyses of the dynamic changes of serum miRNAs in T2DM patients with and without microvascular complications are still lacking currently. In the present study, we used high-throughput TLDA technology to determine a global serum miRNA expression profile in T2DM patients with and without microvascular complications followed by RT-qPCR confirmation in individual samples and successfully identified a profile of five novel miRNAs, including miR-661, miR-571, miR-770-5p, miR-892b and miR-1303, the expression of which was significantly increased in T2DM patient serum. Moreover, levels of these five miRNAs were markedly higher in patients with complications than those without complications. Statistics analysis indicated that the identified miRNAs were closely associated with T2DMC. To our knowledge, this is the first comprehensive study regarding the use of serum miRNAs as useful predictive biomarkers for T2DM-related microvascular complications. We therefore hypothesize that these five circulating miRNAs may serve as novel independent risk factors and may be involved in T2DM pathogenesis and diabetic microvascular complications.

Despite intensive research and rapid progress in recent decades, the major mechanisms underlying T2DM pathogenesis as well as its microvascular complications remain obscure, and a definitive cure is still unavailable^18^. Biomarkers for the early detection of this disease and the identification of individuals at risk of developing complications would greatly improve patient care. However, none of such novel biomarker can avalible and efficiently predict T2DM currently^19^,^20^. Recent studies from our group and others have demonstrated that extracellular miRNAs were stably presented in circulation and can be used as promising diagnostic diabetes biomarkers^11^,^12^,^13^. The first report describing circulating miRNAs in T2DM patients was conducted by Zampetaki and colleagues, who identified a panel of 10 downregulated plasma miRNAs that were useful biomarkers for distinguishing T2DM patients from controls and decreased plasma miR-126 expression was associated with a risk for future diabetes development^21^. This result was replicated by two recent studies describing that circulating miR-126 can be developed into a non-invasive and useful diagnostic tool for predicting individuals who are susceptible to developing T2DM^20^,^22^. Another study found seven diabetes-related miRNAs that were significantly elevated in new onset T2DM compared with pre-diabetic individuals and/or T2DM-susceptible individuals with normal glucose tolerance^23^. The deregulation of serum miRNAs was also associated with T2DM and obesity^24^. However, compared with the above findings, we did not observe overlap in altered miRNAs when comparing our current study with previous studies. We speculate that this inconsistency may be produced not only by sample properties but also by the following factors: 1) miRCURY LNA™ microRNA PCR System and SYBR Green qPCR SuperMix-UDG methodologies were used in previous studies, whereas we performed TaqMan probe-based RT-qPCR to detect miRNA levels in individual serum samples, and 2) different normalization methods were used in these studies. In previous studies, circulating miRNA expression levels were normalized to U6, synthetic cel-miR-39 or unadjusted Ct values, and serum miRNA expression levels were normalized to a spike-in exogenous RNA (MiR2911) in our study. Nevertheless, the heterogeneity of the results still raises an issue of an urgent need for large prospective and multicenter studies to identify reliable miRNA signatures for diagnosing T2DM based on standardized sample preparation, RNA extraction and miRNA data analysis protocols.

The exact reasons and underlying mechanisms of increased serum miRNA levels in T2DM remains to be explored; however, previous studies have indicated that miRNAs can be actively or passively released by a variety of cells. Furthermore, circulating miRNAs are transported by microvesicles, Ago protein complexes or HDL and can be transferred in an active form to recipient cells^25^. Based on these results, we speculated that modifications of our five identified miRNAs may change simultaneously during the course of T2DM and T2DMC, and altered miRNAs originating from specific groups of cells may provide detailed information about the physiological or pathological status of the target tissues in which diabetes complications occur. Of our five identified miRNAs, study revealed that miR-661 participated in the regulation of insulin biogenesis and the SNAIL-triggered epithelial to mesenchymal transition^26^, which indicates that this miRNA may contribute to T2DM and its related microvascular complications. One study demonstrated that miR-571 serum levels were upregulated in patients with chronic liver disease and play putative roles in fibrogenic and inflammatory processes in distinct cellular compartments that are involved in liver cirrhosis pathogenesis^27^. Because fibrosis and the inflammatory response are also key pathological processes involved in diabetic nephropathy, we speculate that miR-571 may contribute to kidney fibrosis and highlight the role of some aspects of the EMT pathway in diabetic nephropathy. Only one study has demonstrated the role of miR-770-5p, and bioinformatic analysis revealed that it likely contributed to apoptosis and hippocampal signaling pathways and was involved in the molecular mechanisms underlying temporal lobe epilepsy memory disorders^28^. Like miR-770-5p, recent study suggested that miR-892b was correlated with retinopathy and neurological diseases^29^,^30^. These results suggest that this miRNA is a potential contributor to diabetic neuropathy and retinopathy. Finally, miR-1303 was mostly reported to be tumor/cell cycle-related miRNAs^31^. Collectively, these findings might open the door to future investigations aimed at assessing the potential use of miRNAs as predictors of T2DM and its associated complications.

One of the weaknesses of the present study is the use of three pooled samples in miRNAs screening phase, because results acquired from pooled samples might yield some inaccurate information due to individual difference and result a decreased specificity of the test, thus initial screening in individual sample still be preferred and recommended in similar study in future. Another notably issue is that the all the samples including the cases and controls were Chinese Han population that were enrolled in one center, therefore, future systematical analyzes of serum miRNA expression profiles from multiethnic and multicentric T2DM cases are needed.

In summary, we have defined a novel five-serum miRNA panel that is associated with T2DM and T2DM-related microvascular complications. Specifically, we demonstrated that the serum levels of miR-661, miR-571, miR-770-5p, miR-892b and miR-1303 were altered in T2DM patients. Furthermore, levels of the above five miRNAs were markedly higher in patients with complications than those free of complications. These results suggest that these miRNAs have potential as novel risk indicators of T2DM and its related complications and may play important roles in T2DM pathogenesis and progression as well as T2DM-associated microvascular complications. Further research is necessary to verify that this miRNA panel is involved in the physiological and pathological processes of T2DM as well as their associated complications.

## Methods

### Study subjects and sampling process

The present study was approved by the Research Ethics Committees of Jinling Hospital in accordance with the Declaration of Helsinki, and written informed consent was obtained from each participant. A total of 276 subjects including 184 T2DM patients and 92 healthy subjects were recruited in this study. All of the T2DM patients who were newly diagnosed or had a previously diagnosed disease but had ceased taking drugs for at least 1 month were randomly enrolled from the Department of Endocrinology, Jinling Hospital, Nanjing, China from September 2013 to March 2015. T2DM was diagnosed according to the World Health Organization criteria (WHO) and was defined as fasting plasma glucose of ≥7.0 mmol/L (126 mg/dl) and/or 2-h of glucose of ≥11.1 mmol/L (200 mg/dl) in the 75-g OGTT and/or HbA1c ≥6.5%. Of the 184 patients, 92 were diagnosed with microvascular complications (2 with diabetic foot, 31 with diabetic retinopathy, 32 with diabetic neuropathy and 7 with diabetic nephropathy; the others had a combination of complications), and the other 92 T2DM patients were without clinical signs and symptoms of any complications. The mean diabetes duration were 2.1 ± 2.7 years and 4.8 ± 4.2 years for T2DM patients and T2DMC patients, respectively. All microvascular complications were documented by medical records. Briefly, diabetic foot was defined by T2DM patients presented with obvious diabetic foot ulceration, infection, erythema, pain and purulent drainage, or destruction of deep tissues of the foot associated with neuropathy and/or peripheral arterial disease in the lower extremity. Diabetic nephropathy was diagnosed with serum creatinine level equal or greater than 2 mg/dL, or positive dipstick proteinuria, or the presence of random micro albuminuria/creatinine ratio greater than 30 mg/g. Diabetic retinopathy was diagnosed by detailed fundus examination, including either proliferative or background diabetic retinopathy diagnosed by an ophthalmologist. Diabetic neuropathy was defined as the presence of symptoms and/or signs of peripheral nerve dysfunction in people with diabetes after the exclusion of other causes. Participants were excluded from the study if they had type 1 diabetes mellitus, other types of diabetes, a severe infection, acute cerebrovascular disease, or recent surgery. The recruitment of 92 healthy subjects to the parallel control group was also conducted in the Healthy Physical Examination Center of the Jinling Hospital. The health checkup included a detailed history, physical examination and blood test. Blood was collected in serum vacutainer tubes with a clot activator from participants at least 12 h following their most recent meal, and serum was immediately separated by a 10-min centrifugation at 1500 g. Serum glucose levels were examined using the autoanalyzer method (Hitachi 7600, Hitachi High-Technologies Corporation, Tokyo, Japan). Other clinical biochemical parameters including TC, TG, LDL-C, and HDL-C levels were also measured. The remaining serum samples were stored at −80 °C until miRNA analysis.

### RNA isolation, TaqMan Low Density Array and RT-qPCR assay

A multiphase, case-control study was designed to systematically characterize the serum miRNA expression profile for T2DM patients with and without microvascular complications (Fig. 1). RNA isolation, TaqMan Low Density Array and RT-qPCR assay were performed as previously described^32^. More details can be seen in Supplementary Methods.

### Serum biochemical parameter determination

Serum TC, TG, LDL and HDL concentrations were measured using commercial reagents (RANDOX) on a Hitachi 7600 analyzer (Hitachi High-Technologies Corporation, Tokyo, Japan). HbA1C were also assessed using commercial reagents (TOSOH Corporation) on a HLC-723GB autoanalyzer (TOSOH Corporation, Tokyo, Japan).

### Statistical analysis

Statistical analysis was performed with SPSS 16.0 software. miRNA data are represented as the means ± SEM, and the other variables are expressed as the means ± SD. The nonparametric Mann–Whitney U-test was used to compare the differences in the miRNA concentrations among the groups. Student’s t-test or two-sided χ^2^ test was used to compare the differences in other variables among the groups. A p value < 0.05 was considered to be statistically significant. Forward stepwise binary logistic regression analysis was also conducted to evaluate the influences of serum miRNAs on T2DM and T2DMC, controlling for other variables. Pearson correlation analysis was conducted to evaluate the correlations of miRNA profiles between the three groups. Spearman rank correlation test was used to calculate the associations between miRNAs and clinical biochemical parameters.

## Additional Information

**How to cite this article**: Wang, C. *et al.* Increased serum microRNAs are closely associated with the presence of microvascular complications in type 2 diabetes mellitus. *Sci. Rep.* **6**, 20032; doi: 10.1038/srep20032 (2016).

## Supplementary Material

Supplementary Information

## Figures and Tables

**Table 1:** Markedly upregulated miRNAs in pooled plasma samples from the T2DM and T2DMC groups compared with the non-diabetic control group as determined by TLDA.^a^

miRNA	Control		T2DM			T2DMC		Fold change (T2DMC/Control)
	Cq	Avg Delta Cq	Cq	Avg Delta Cq	Fold change (T2DM/Control)	Cq	Avg Delta Cq	
miR-661	Undetermined	25.13	23.99	7.10	267949.76	23.85	8.11	133649.51
miR-571	Undetermined	25.13	25.98	9.10	67264.52	24.05	8.30	116611.62
miR-1267	Undetermined	25.13	27.05	10.16	32127.12	24.93	9.18	63364.67
miR-564	Undetermined	25.13	25.98	9.10	67123.95	25.11	9.36	55871.89
miR-1208	Undetermined	25.13	28.04	11.15	16179.34	27.02	11.27	14855.41
miR-664	Undetermined	25.13	27.00	10.11	33204.54	27.96	12.22	7741.79
miR-584	Undetermined	25.13	28.04	11.16	16112.37	27.99	12.24	7589.76
miR-1303	Undetermined	25.13	28.99	12.10	8378.93	28.07	12.33	7159.14
miR-770-5p	Undetermined	25.13	28.78	13.12	4148.51	28.95	13.20	3900.61
miR-892b	Undetermined	25.13	30.99	14.10	2095.04	29.00	13.26	3759.46
miR-886-5p	Undetermined	25.11	27.98	11.08	16698.56	29.99	14.28	1822.19
miR-212	30.99	16.10	31.99	15.08	2.02	21.13	5.42	1634.44
miR-516-3p	31.96	17.09	29.96	13.07	16.22	28.97	13.22	14.66
miR-1255b-5p	31.03	16.16	30.97	14.09	4.21	28.98	13.23	7.62
miR-30a-3p	30.00	15.13	26.97	10.08	33.10	27.95	12.21	7.58
miR-151a-5p	30.01	15.14	26.99	10.11	32.79	28.00	12.25	7.41
miR-30e-3p	29.95	15.08	26.95	10.06	32.43	27.94	12.20	7.40
miR-378a-3p	31.03	16.16	28.93	12.05	17.33	29.98	14.24	3.80
miR-638	30.03	15.16	28.94	12.06	8.58	29.01	13.26	3.73
miR-1227	28.00	13.13	27.99	11.10	4.08	26.98	11.24	3.71
miR-605	28.00	13.13	26.95	10.06	8.39	26.99	11.25	3.68
miR-132-3p	27.99	13.10	25.99	9.09	16.12	26.99	11.28	3.54
miR-1260a	21.94	7.07	22.95	6.07	2.01	21.00	5.25	3.52
miR-652-3p	30.99	16.09	27.01	10.11	63.47	29.99	14.28	3.52
miR-15b-5p	28.97	14.08	25.98	9.08	31.94	27.98	12.27	3.51

^a^“undetermined” is referred as the Cq value of 40 when the fold change is calculated. T2DM = type 2 diabetes. T2DMC = type 2 diabetes with microvascular complications.TLDA = TaqMan Low Density Array.

**Table 2:** Demographic and clinical features of the T2DM, T2DMC and non-diabetic control group.^a^

Variable	Controls (n = 92)	T2DM (n = 92)	p value^b^	T2DMC (n = 92)	p value^b^	p value^c^
Age (years)	50.2 (14.2)	47.7 (13.9)	0.241	52.4 (13.1)	0.257	0.019
Sex- no.(%)			0.761^d^		0.026^d^	0.055^d^
Male	56 (60.9)	58 (63.0)		70 (76.1)		
Female	36 (39.1)	34 (37.0)		22 (23.9)		
BMI (kg/m^2^)	23.6 (2.0)	25.6 (4.5)	<0.001	25.5 (2.9)	<0.001	0.841
Somking status- no.(%)			0.854^d^		0.063^d^	0.093^d^
Ever and Current	18 (19.6)	19 (20.7)		29 (31.5)		
Never	74 (80.4)	73 (79.3)		63 (68.5)		
Alcohol consumption-no.(%)			0.058^d^		0.862^d^	0.084^d^
Ever and Current	22 (23.9)	12 (12.0)		21 (22.8)		
Never	70 (76.1)	80 (88.0)		71 (77.2)		
SBP (mmHg)	122.1 (6.8)	134.1 (16.5)	<0.001	135.5 (14.4)	<0.001	0.534
DBP (mmHg)	78.3 (4.1)	81.2 (10.3)	0.012	82.5 (11.1)	0.001	0.435
Diabetic duration (years)		2.1 (2.7)		4.8 (4.2)		
Glucose (mmol/L)	4.9 (0.5)	9.2 (4.3)	<0.001	9.5 (3.4)	<0.001	0.645
HbA_1c_ (percent)	5.3 (0.4)	9.8 (2.9)	<0.001	10.3 (2.7)	<0.001	0.254
Total cholesterol (mmol/L)	4.4 (0.5)	9.2 (4.3)	<0.001	9.5 (3.7)	<0.001	0.645
Triglyceride (mmol/L)	0.9 (0.3)	2.6 (2.7)	<0.001	2.1 (1.6)	<0.001	0.113
LDL-C (mmol/L)	2.5 (0.6)	2.7 (0.8)	0.016	2.9 (0.9)	<0.001	0.125
HDL-C (mmol/L)	1.4 (0.3)	0.97 (0.2)	<0.001	1.0 (0.2)	<0.001	0.362
Complications						
Diabetic retinopathy				31 (33.7)		
Diabetic neuropathy				32 (34.8)		
Diabetic nephropathy				7 (7.6)		
Diabetic foot				2 (2.2)		
Combined				20 (21.7)		

^a^Data are mean (SD) or number (%).

^b^Compared with control group.

^c^Compared between the two case group.

^d^Two sides χ^2^ test. T2DM = type 2 diabetes. T2DMC = type 2 diabetes with microvascular complications. BMI = body mass index. SBP = systolic blood pressure. DBP = diastolic blood pressure. LDL-C = low density lipoprotein cholesterol. HDL-C = high density lipoprotein cholesterol.

**Table 3:** Univariate and multivariate logistic regression analyses of serum miRNAs for T2DM and T2DMC.

miRNA	miR-571		miR-661		miR-770-5p		miR-892b		miR-1303		miR-Panel	
	Relative risk (95% CI)	p value	Relative risk (95% CI)	p value	Relative risk (95% CI)	p value	Relative risk (95% CI)	p value	>Relative risk (95% CI)	p value	Relative risk (95% CI)	p value
Univariate analyses												
Model 1^a^												
T2DM	7.6 (2.8–20.8)	<0.001	11.2 (4.1–30.2)	<0.001	9.7 (3.6–26.4)	<0.001	8.0 (2.9–21.8)	<0.001	5.5 (2.0–15.2)	<0.001	7.2 (3.5–14.8)	<0.001
T2DMC	19.8 (7.4–53.4)	<0.001	25.9 (9.6–69.8)	<0.001	22.6 (8.4–60.9)	<0.001	19.8 (7.4–53.3)	<0.001	16.7 (6.2–44.8)	<0.001	16.3 (7.7–34.3)	<0.001
Model 2^b^												
T2DMC	2.6 (1.4–4.8)	0.002	2.3 (1.3–4.2)	0.005	2.3 (1.3–4.2)	0.005	2.5 (1.4–4.5)	0.003	3.0 (1.6–5.7)	0.001	2.3 (1.2–4.2)	0.01
Multivariate analyses (adjusted for age, sex, BMI, smoking status, alcohol consumption habits, SBP and DBP)												
Model 1^a^												
T2DM			6.9 (2.0–24.2)	0.003			7.2 (1.6–32.0)	0.009				
T2DMC	11.0 (2.8–43.2)	0.001	19.7 (5.5–70.6)	<0.001								
Model 2^b^												
T2DMC									3.3 (1.7–6.4)	<0.001		

^a^Model 1: the reference category was the control group.

^b^Model 2: the reference category was the T2DM group. BMI = body mass index. SBP = systolic blood pressure. DBP = diastolic blood pressure.
