# Supplementary material for: Increased serum microRNAs are closely associated with the presence of microvascular complications in type 2 diabetes mellitus
Source: Sci Rep. 2016 Feb 1;6:20032. doi: 10.1038/srep20032 (PMC4735518; doi:10.1038/srep20032)
Supplement: Supplementary Information [file srep20032-s1.doc]

**Increased serum microRNAs are closely associated with the presence of microvascular complications in type 2 diabetes mellitus**

Cheng Wang^1,2,†^, Shujun Wan^1,†^, Ting Yang^1,2,†^, Dongmei Niu^1^, Aisen Zhang^3^, Cuihua Yang^4^, Jialu Cai^1^, Jia Wu^1^, Jiaxi Song^1^, Chen-Yu Zhang^2,*^, Chunni Zhang^1,2,*^ & Junjun Wang^1,2,*^

^1^Department of Clinical Laboratory, Jinling Hospital, Nanjing University School of Medicine, Nanjing, 210002, China. ^2^ State Key Laboratory of Pharmaceutical Biotechnology, Nanjing Advanced Institute for Life Sciences, Nanjing University School of Life Sciences, Jiangsu Engineering Research Center for MicroRNA Biology and Biotechnology, Nanjing University, Nanjing, 210046, China; ^3^Department of Geratology, the First Hospital Affiliated to Nanjing Medical University, Nanjing, 210029, China.^4^Department of Endocrinology, Jinling Hospital, Nanjing University School of Medicine, Nanjing, 210002, China.

Correspondence and requests for materials should be addressed to J.W. (email: wangjunjun9202@163.com) or C.Z. (email: zchunni27@hotmail.com) or C.Y.Z (email: cyzhang@nju.edu.cn)

^†^ These authors contributed equally to this work.

**Keywords:** type 2 diabetes mellitus, serum microRNAs, microvascular complications, biomarker, risk factor

**Supplementary information**

**Supplementary Methods**

**RNA isolation,** **TaqMan Low Density Array and RT-qPCR assay**

For the initial TaqMan Low Density Array (TLDA) screening stage, equal volumes of serum (500 μL each) from 24 patients with T2DM, 24 patients with T2DM microvascular complications and 24 non-diabetic controls were pooled separately to form case and control sample pools. Total RNA was extracted from each pooled serum sample (each pool contained 12.0 mL) using Trizol reagent (Invitrogen) according to the manufacturer’s instructions with minor modifications as previously described^32^. The resulting RNA pellet was dissolved in 20 μL DEPC-treated water and stored at −80°C until further analysis. The miRNA profile of 754 different human miRNAs (TaqMan Array Human MicroRNA A+B Cards Set v3.0) (Life Technologies, Carlsbad, CA, USA) was then examined using TLDA on an ABI PRISM 7900HT. miRNA expression levels are presented as threshold cycle (Cq) values and normalized to endogenous U6 in each pools as recommended by the manufacturer. Relative content was calculated using the comparative Cq method (2^-ΔΔCq^).

For the serum RT-qPCR assay, total RNA was extracted from 100 µL serum with a 1-step phenol/chloroform purification protocol as previously described^32^. A hydrolysis probe-based RT-qPCR assay was performed according to the manufacturer’s instructions (7500 Sequence Detection System, Applied Biosystems) with a minor modification as previously described^30^. All reactions including no-template controls were conducted in triplicate. To control for variability in RNA extraction and purification procedures, an exogenous plant miRNA MIR2911 (5’-GGCCGGGGGACGGGCUGGGA-3’) was spiked into each sample with a final concentration of 10^6^ fmol/L during RNA isolation as a synthetic external reference for serum miRNA normalization. Relative levels of targeted miRNAs were then normalized to MIR2911 and were calculated using the comparative Cq method (2^-ΔΔCq^). ΔCq was calculated by subtracting the Cq values of MIR2911 from the Cq values of the target miRNAs. 2^-ΔΔCq^ was then determined by the average 2^-ΔCq^ values of the cases group divided by the average 2^-ΔCq^ values of the control group.

**Risk score analysis**

A risk score analysis was performed to calculate the risk score function (RSF) for patient and control samples samples with the purpose of evaluating the associations between serum-miRNA panel and T2DM. In brief, the risk score of each miRNA, denoted as *s*, was set to 1 if its concentration was higher than the upper 95% reference interval for the corresponding miRNA concentration in controls and to 0 otherwise. A risk score function to predict NSCLC was defined according to a linear combination of concentration for each miRNA. For example, the RSF for sample *i* using information from five miRNAs was: rsf*_i_*=∑^5^*_j-1_*W*_j_*.s*_ij_*. In the above equation, s*_ij_* is the risk score for miRNA *j* on sample *i*, and W*_j_* is the weight of the risk score of miRNA *j*. To determine the W*_s_*, five univariate logistic regression models were fitted using the disease status with each of the risk scores. The regression coefficient of each risk score was used as the weight to show the contribution of each miRNA to the RSF.

**Supplementary tables and figures**

**
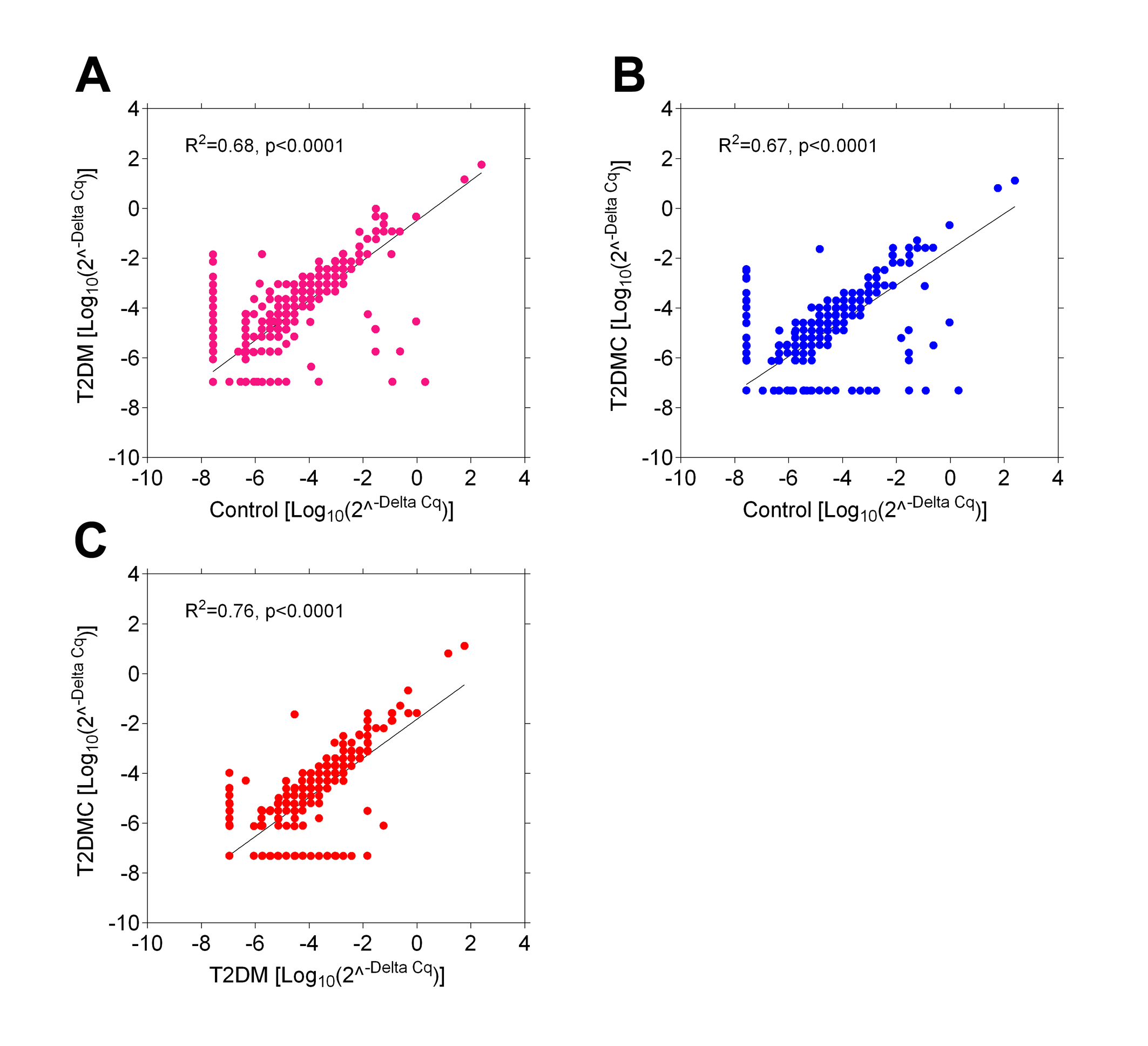
**

**Supplementary Figure S1. Pearson correlation scatter plot of serum miRNA profiles (754 miRNAs) of the T2DM, T2DMC and non-diabetic control groups as determined by TLDA.**

**
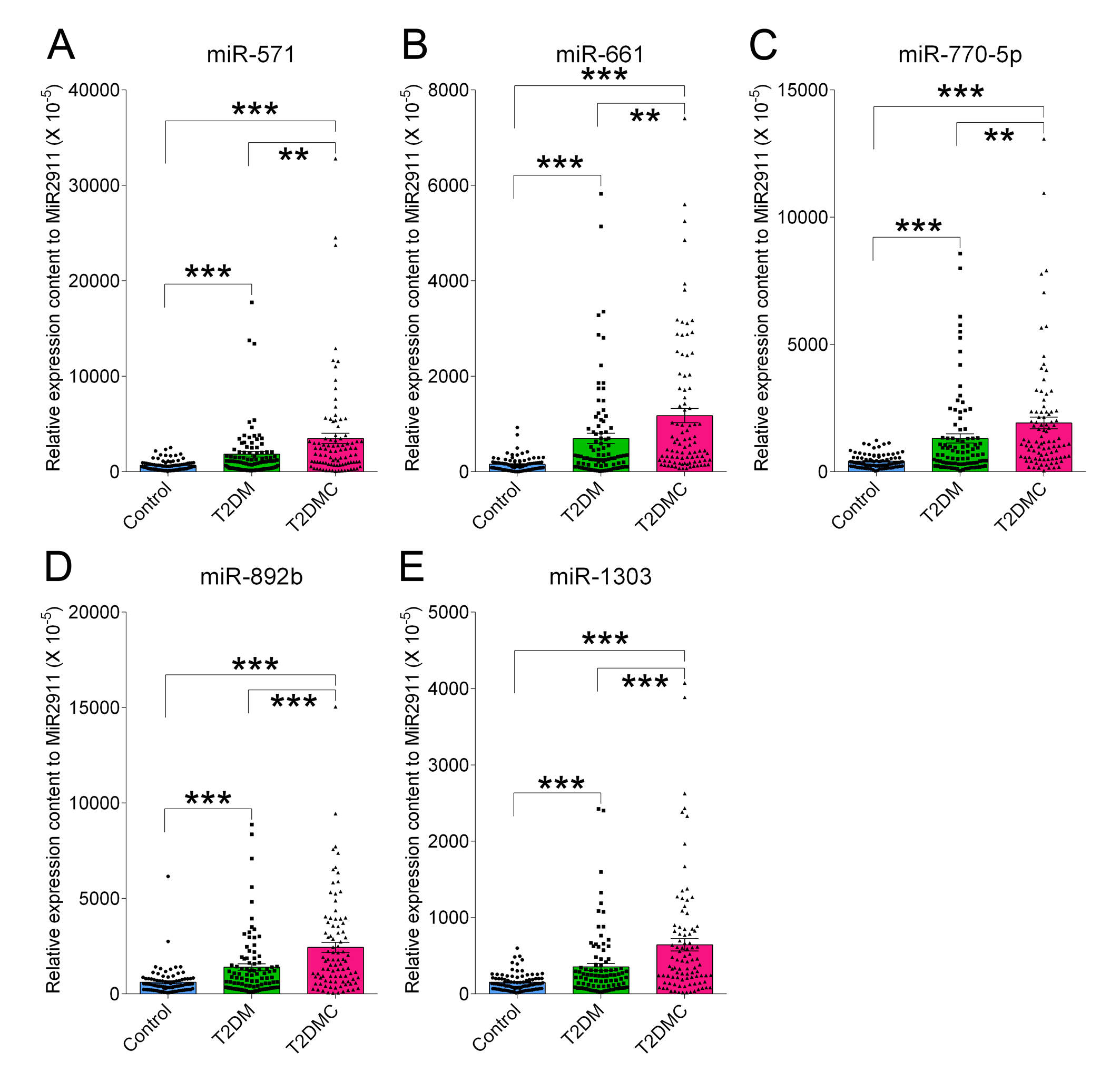
**

**Supplementary Figure S2. Differential expression of the five miRNAs (A) miR-571, (B) miR-661, (C) miR-770-5p, (D) miR-892b and (E) miR-1303 in the serum samples of T2DM (n=92), T2DMC (n=92) and controls (n=92) enrolled in the training set and validation set.** The relative levels of the five miRNAs to MIR2911 were detected by RT-qPCR individually. Each point represents the mean of triplicate samples. Each p-value was derived from a nonparametric Mann–Whitney U-test. **p < 0.01; ***p < 0.001.

**
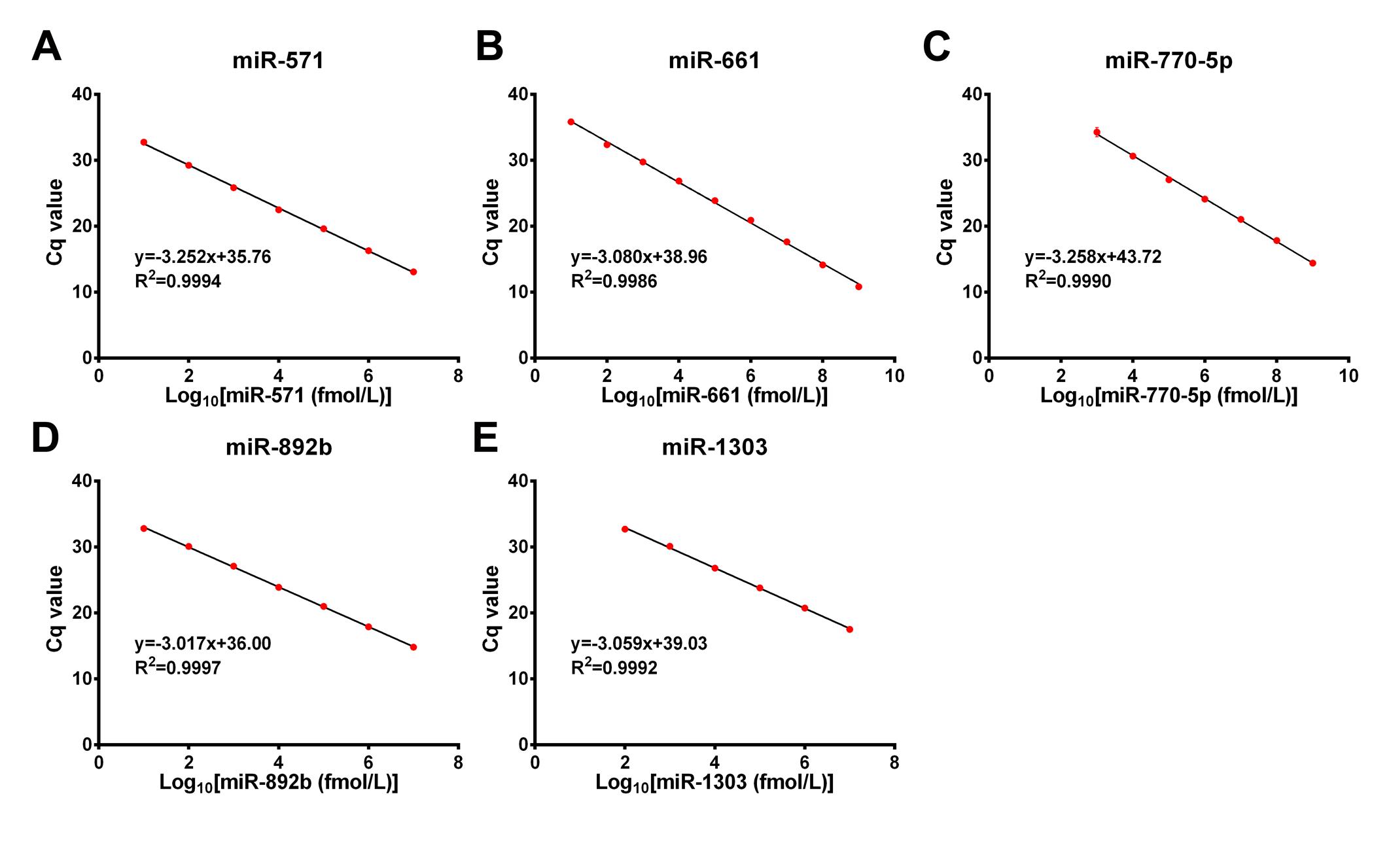
**

**Supplementary Figure S3.** **Standard curves of miR-571, miR-661, miR-770-5p, miR-892b and miR-1303 using synthetic mature miRNAs (A-E)**. For each assay, ten-fold serial dilution of synthetic miRNA from 10 fmol/L to 10^7^ pmol/L was used to generate the standard curves. The resulting Cq values were plotted versus the log_10_ of the amount of synthetic miRNAs. Each point represents the mean of three independent experiments.


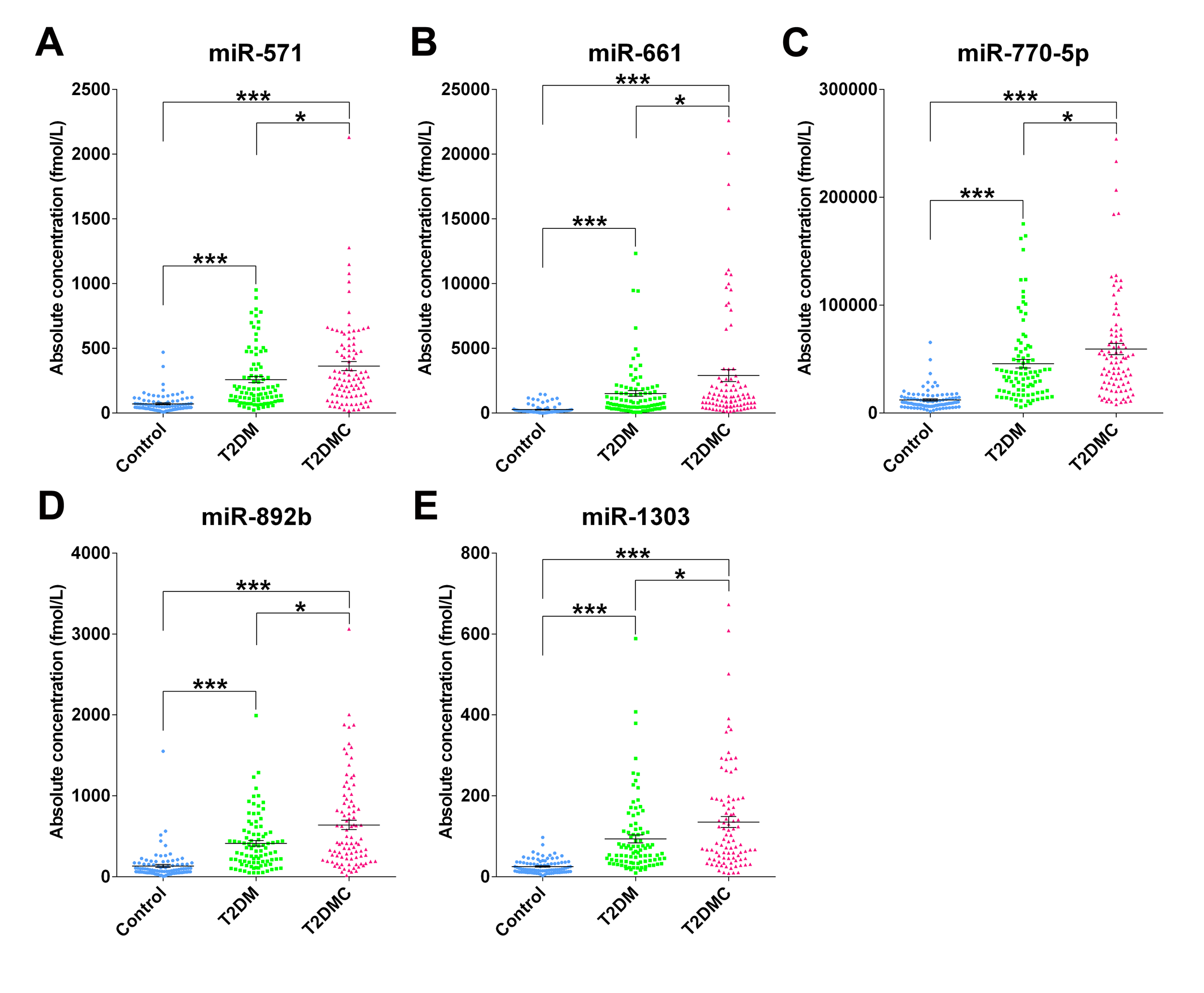


**Supplementary Figure S4. Absolute concentrations of the five identified serum miRNAs (A) miR-571, (B) miR-661, (C) miR-770-5p, (D) miR-892b and (E) miR-1303 in the T2DM, T2DMC and non-diabetic control groups of training set and validation set.** Cq values were converted to absolute concentrations using the corresponding calibration curves. Each point represents the mean of triplicate samples. *p < 0.05; ***p < 0.0001.


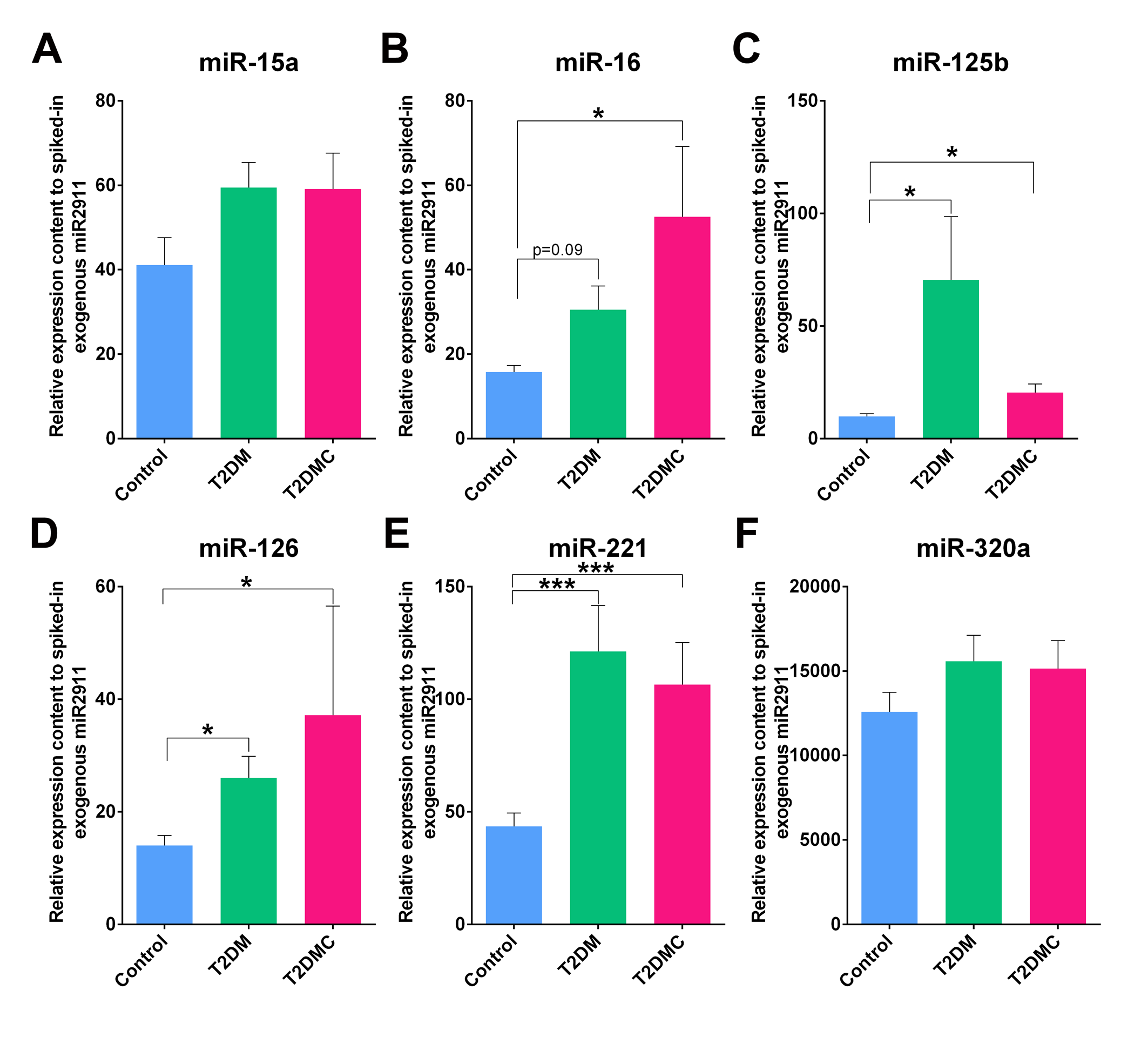


**Supplementary Figure S5.** **Relative levels of six serum miRNAs (A) miR-15a, (B) miR-16, (C) miR-125b, (D) miR-126, (E) miR-221 and (F) miR-320a that selected from literature in 31 T2DM, 31 T2DMC patients, and 31 non-diabetic control groups of validation set.** Cq values were converted to relative concentrations normalized to MIR2911 values and were calculated using the comparative Cq method (2^-ΔCq^). Each point represents the mean of triplicate samples. *p < 0.05; ***p < 0.0001.


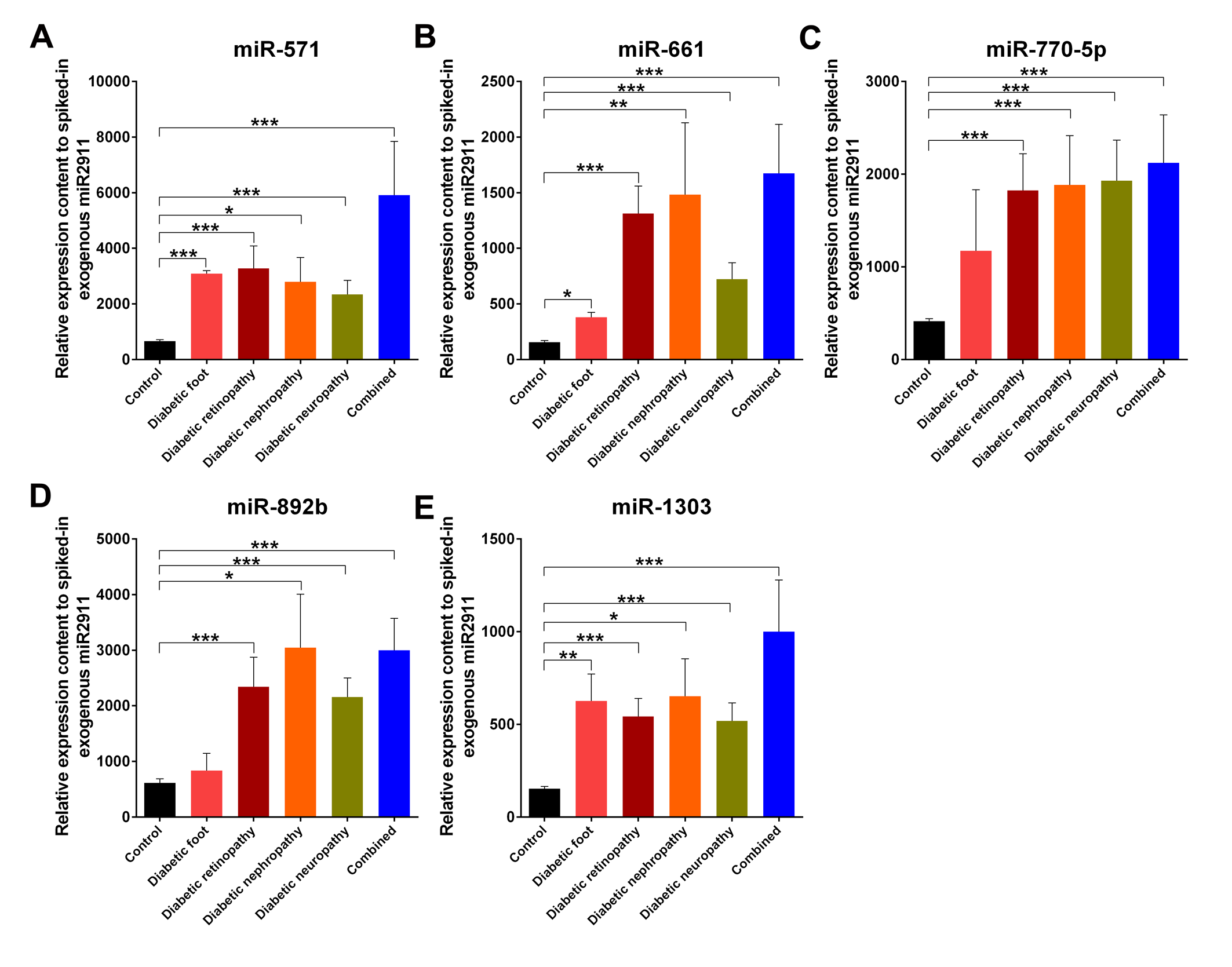


**Supplementary Figure S6. Relative levels of the five identified serum miRNAs (A) miR-571, (B) miR-661, (C) miR-770-5p, (D) miR-892b and (E) miR-1303 in the T2DMC patients represented with diabetic foot, retinopathy, neuropathy, nephropathy, combination complications, and non-diabetic control groups of training set and validation set.** Cq values were converted to relative concentrations normalized to MIR2911 values and were calculated using the comparative Cq method (2^-ΔCq^). Each point represents the mean of triplicate samples. *p < 0.05;** p <0.01; ***p < 0.0001.

**Supplementary Table S1. Raw Cq value and relative concentrations to MIR2911 of the miRNA in the serum samples from T2DM, T2DMC and non-diabetic control groups determined by individual RT-qPCR assay in training set.a**

| **miRNA** | **Control** | | **T2DM** | | **Change (T2DM/Control)** | | **T2DMC** | | **Change (T2DM/Control)** | | **Background (negative control)** |
| --- | --- | --- | --- | --- | --- | --- | --- | --- | --- | --- | --- |
|  | **Raw Cq** | **Relative levels** | **Raw Cq** | **Relative levels** | **Fold** | **p value^b^** | **Raw Cq** | **Relative levels** | **Fold** | **p value^b^** | **Raw Cq** |
| miR-661 | 30.2±0.5 | 181.1±42.2 | 26.5±0.3 | 1173.4±181.1 | 6.5 | <0.001 | 25.7±0.4 | 2023.2±314.5 | 11.2 | <0.001 | 37.2±1.6 |
| miR-571 | 27.9±0.2 | 598.4±115.6 | 25.7±0.2 | 1970.6±299.4 | 3.3 | <0.001 | 25.0±0.3 | 3280.7±545.5 | 5.5 | <0.001 | 35.7±1.9 |
| miR-1267 | 22.3±0.3 | 21625.4±4258.0 | 20.8±0.4 | 63099.3±16128.7 | 2.9 | <0.01 | 19.8±0.4 | 100290±13649.5 | 4.6 | <0.001 | 25.1±0.5 |
| miR-564 | 26.4±0.2 | 1249.3±273.3 | 25.2±0.2 | 2096.4±246.7 | 1.7 | <0.01 | 24.5±0.2 | 3596.2±422.7 | 2.9 | <0.001 | 26.5±0.3 |
| miR-1208 | 27.9±0.2 | 453.4±90.1 | 27.1±0.2 | 667.0±91.6 | 1.5 | <0.05 | 26.4±0.3 | 1206.6±191.8 | 2.7 | <0.01 | 32.3±0.4 |
| miR-664 | 26.0±0.2 | 1726.0±346.4 | 24.1±0.3 | 4114.2±598.3 | 2.4 | <0.001 | 23.6±0.1 | 6480.9±604.6 | 3.8 | <0.001 | 27.8±0.3 |
| miR-584 | 28.7±0.3 | 256.2±51.4 | 27.2±0.2 | 576.7±60.7 | 2.3 | <0.001 | 26.5±0.3 | 917.2±100.1 | 3.6 | <0.001 | 31.3±0.5 |
| miR-1303 | 29.9±0.2 | 154.5±30.5 | 27.9±0.2 | 423.7±69.4 | 2.7 | <0.001 | 27.1±0.3 | 569.0±80.7 | 3.7 | <0.001 | Undetermined |
| miR-770-5p | 28.4±0.3 | 372.6±54.7 | 26.4±0.2 | 1198.2±241.7 | 3.2 | <0.001 | 25.3±0.2 | 1970.1±279.4 | 5.3 | <0.001 | 34.3±0.3 |
| miR-892b | 27.8±0.4 | 796.2±260.2 | 25.8±0.2 | 1618.7±220.2 | 2.03 | <0.001 | 25.0±0.1 | 2556.5±355.1 | 3.2 | <0.001 | 33.6±0.2 |
| miR-886-5p | 26.5±0.4 | 1708.3±399.0 | 25.6±0.3 | 1807.3±223.6 | 2.3 | 0.093 | 25.2±0.3 | 2550.1±451.4 | 3.6 | 0.101 | 27.8±0.3 |
| miR-212 | 25.4±0.3 | 2405.7±450.1 | 24.1±0.1 | 4707.0±546.7 | 1.95 | <0.01 | 23.3±0.1 | 8416.3±886.6 | 3.5 | <0.001 | 26.3±0.4 |
| miR-516-3p | 27.4±0.2 | 1011.5±225.1 | 26.0±0.2 | 1623.0±309.1 | 1.6 | <0.01 | 25.4±0.3 | 2431.1±469.7 | 2.4 | <0.001 | 31.1±0.4 |

**^a^** Data are mean (SEM).^b^ Compared with control group. T2DM= type 2 diabetes. T2DMC= type 2 diabetes with microvascular complications.Cq= quantification cycle

**Supplementary Table S2. ROC curves for the capacity of the serum miRNAs and their panel to differentiate T2DM or T2DMC cases from control individuals and to differentiate T2DMC from T2DM patients**.

| **miRNA** | **T2DM vs Control** | | | | | **T2DMC vs Control** | | | | | **T2DM vs T2DMC** | | | | |
| --- | --- | --- | --- | --- | --- | --- | --- | --- | --- | --- | --- | --- | --- | --- | --- |
|  | **AUC** | **SEM** | **p value** | **95% CI** | | **AUC** | **SEM** | **p value** | **95% CI** | | **AUC** | **SEM** | **p value** | **95% CI** | |
|  |  |  |  | **Lower Bound** | **Upper Bound** |  |  |  | **Lower Bound** | **Upper Bound** |  |  |  | **Lower Bound** | **Upper Bound** |
| **miR-571** | 0.70 | 0.04 | < 0.0001 | 0.62 | 0.77 | 0.80 | 0.03 | < 0.0001 | 0.73 | 0.87 | 0.62 | 0.04 | 0.004 | 0.54 | 0.70 |
| **miR-661** | 0.74 | 0.04 | < 0.0001 | 0.66 | 0.81 | 0.87 | 0.03 | < 0.0001 | 0.82 | 0.92 | 0.64 | 0.04 | 0.002 | 0.56 | 0.72 |
| **miR-770-5p** | 0.70 | 0.04 | < 0.0001 | 0.62 | 0.78 | 0.84 | 0.03 | < 0.0001 | 0.79 | 0.90 | 0.63 | 0.04 | 0.002 | 0.55 | 0.71 |
| **miR-892b** | 0.69 | 0.04 | < 0.0001 | 0.61 | 0.77 | 0.81 | 0.03 | < 0.0001 | 0.74 | 0.87 | 0.65 | 0.04 | 0.001 | 0.57 | 0.73 |
| **miR-1303** | 0.67 | 0.04 | < 0.0001 | 0.59 | 0.75 | 0.79 | 0.04 | < 0.0001 | 0.72 | 0.86 | 0.64 | 0.04 | 0.001 | 0.56 | 0.72 |
| **miR-Panel** | 0.71 | 0.04 | < 0.0001 | 0.64 | 0.79 | 0.83 | 0.03 | < 0.0001 | 0.77 | 0.89 | 0.65 | 0.04 | 0.001 | 0.57 | 0.73 |

**Supplementary Table S3. Spearman rank correlations between identified serum miRNAs and sera indices in all the studied samples**.

| **Variables** | **r/p** | **Glu** | **TC** | **TG** | **HDL-C** | **LDL-C** | **HbA1c** |
| --- | --- | --- | --- | --- | --- | --- | --- |
| **miR-571** | *r* | 0.291 | -0.072 | 0.174 | -0.312 | 0.097 | 0 |
|  | p | <0.0001 | 0.233 | 0.004 | <0.0001 | 0.108 | 0.998 |
| **miR-661** | *r* | 0.327 | -0.068 | 0.170 | -0.286 | 0.073 | 0.009 |
|  | p | <0.0001 | 0.261 | 0.005 | <0.0001 | 0.226 | 0.903 |
| **miR-770-5p** | *r* | 0.337 | -0.103 | 0.160 | -0.322 | 0.05 | 0.011 |
|  | p | <0.0001 | 0.088 | 0.008 | <0.0001 | 0.406 | 0.883 |
| **miR-892b** | *r* | 0.293 | -0.065 | 0.179 | -.292 | 0.058 | 0 |
|  | p | <0.0001 | 0.283 | 0.003 | <0.0001 | 0.341 | 0.999 |
| **miR-1303** | *r* | 0.239 | -0.115 | 0.141 | -0.312 | 0.042 | -0.033 |
|  | p | <0.0001 | 0.056 | 0.019 | <0.0001 | 0.492 | 0.649 |

Glu=glucose. TC=Total cholesterol. TG=Triglyceride. LDL-C= low density lipoprotein cholesterol. HDL-C= high density lipoprotein cholesterol.
